# Help-seeking behaviours among cannabis consumers in Canada and the United States: Findings from the international cannabis policy study

**DOI:** 10.1016/j.dadr.2024.100306

**Published:** 2024-12-11

**Authors:** Samantha M. Rundle, David Hammond

**Affiliations:** School of Public Health Sciences, University of Waterloo, ON N2L 3G1 Canada

**Keywords:** Cannabis, Help-seeking, Source of help, Cannabis treatment, Marijuana

## Abstract

**Background:**

Little literature exists on what sources of help individuals utilize for cannabis-related problems. The current study examined the percentage of consumers who sought help to manage cannabis-related problems, such as perceived cannabis use disorder, the most common sources of help sought, and factors associated with help-seeking.

**Methods:**

Past 12-month cannabis consumers (*N* = 13,209) completed an online survey from the International Cannabis Policy Study. Past 3-month help-seeking behaviours, respondent’s perceived addiction to cannabis, legal status of cannabis in their jurisdiction, and risky behaviours associated with cannabis use was assessed.

**Results:**

A minority sought help from any source (9.2 %) with the most likely being a doctor/physician (44.9 %). Help-seekers were most likely to be younger, mixed race (*p* = .011), more educated, financially stable, male, and higher perceived addiction to cannabis (all contrasts *p* < .001). In comparison to consumers in Canada and ‘legal’ US states, respondents in ‘illegal’ US states were more likely to seek help from family and friends (Canada: AOR = 5.73, 2.21–14.91; US: AOR = 4.76, 2.00–11.11) and less likely to seek help from a doctor/physician (Canada: AOR = 0.46, 0.24–0.90; US: AOR = 0.51, 0.27–0.99).

**Conclusion:**

Roughly 1 in 10 cannabis consumers sought help from a range of sources, including a third who are at high risk of problematic use. More informal sources of help, such as seeking help from online sources are frequently used. Future research should examine these frontline sources of help for cannabis consumers.

## Highlights

• Roughly 1 in 10 cannabis consumers in the United States and Canada sought help for their cannabis use in the past year.
• 1 in 3 consumers report some experiencing problematic cannabis use, such as strong desires to use, heavy, frequent use and cannabis use disorder.
• A range of formal and informal sources are being accessed, including doctors, psychologists, online resources and help from family and friends.
• Doctors and physicians were the most sourced source of help followed by online websites.

## 1 Background

In Canada, cannabis use accounts for roughly 5 % (or $2.4 billion) of the costs associated with substance use disorders, including healthcare costs, lost productivity, criminal justice costs and other direct costs such as motor vehicle damages (CCSA, 2023). Approximately one-third of daily consumers meet the criteria for cannabis use disorder (Connor et al., 2021, United Nations, 2020); however, it is estimated that only 10 % of daily or near daily consumers will ever seek treatment for their use (Sherman and McRae-Clark, 2016). Numerous studies have examined barriers to help-seeking among cannabis consumers (Fernández-Artamendi et al., 2012, Kerridge et al., 2017), but little is known about which sources of help individuals are most likely to utilize and the individual-level factors associated with seeking help to manage problems from cannabis use.

The Andersen model identifies three types of factors that impact the use of healthcare services: predisposing, enabling/impeding and need factors (Andersen, 2008). Predisposing factors are sociodemographic factors such as sex, age, race/ethnicity, and education. Enabling/impeding factors include factors that may allow or restrict an individual to seek the treatment they need, such as occupational status, perceived income adequacy, and stigma. Lastly, ‘need factors’ highlight the potential need for treatment such as cannabis use consequences, frequency of cannabis use, and age at onset of cannabis use. A recent systematic review reports higher needs are associated with higher healthcare use (Hajek et al., 2021).

The severity of an individual’s substance use, negative attitudes toward treatment, not wanting to stop using the substance, and fear of stigmatization are all well established barriers to the treatment for addictive disorders in general (Chen et al., 2013, Vayr et al., 2019), including cannabis use disorder (Kerridge et al., 2017, Van Der Pol et al., 2013). Although treatment seeking barriers are well documented, little research has focused on what sources of help-seeking individuals’ access. To date, most research in this area has examined treatment seeking for formal addiction support services, including inpatient and outpatient drug dependency programs (Halladay et al., 2019, Olthof et al., 2022, Van Der Pol et al., 2013). A large (*N* = 36,309) national survey of US adults found that individuals who were previously married, in the lowest income category, had a family history of drug problems, and greater cannabis-use disorder severity, along with other mental health diagnoses were most likely to seek formal addiction treatment services (Kerridge et al., 2017). Similarly, Van Der Pol et al. (2013) and Mongan et al. (2022) found that treatment seekers were more likely to be those who initiated cannabis use at a younger age, use cannabis in greater quantities, display more cannabis dependence characteristics, have less social support, more pressure to seek treatment, be unemployed and have lower educational attainment.

In contrast, there is little research on other sources of help-seeking such as discussing the management of cannabis use problems with doctors or physicians, psychiatrists or psychologists or even help-seeking from less formal, but more accessible sources of help, including online sources, smartphone applications, or support from family and friends. A recent survey of Dutch cannabis consumers investigated the appeal of different types of help-seeking strategies among frequent cannabis consumers including books, online forums, social support, general practitioners, outpatient or inpatient substance use disorder program, or self-help groups (Olthof et al., 2022). The most appealing sources of support were social support, online sources, outpatient treatment programs and support from a general practitioner. Within this study, four subgroups of cannabis consumers were identified based on these help-seeking preferences. The largest subgroup (over half of their sample) was grouped into the “no support/only social” group who only found social support appealing. The second largest subgroup, the “GP/outpatient” group, found their general practitioner and outpatient substance use disorder treatment programs most appealing, followed by the “online help” subgroup and “all sources” subgroup which identified that online help and all sources of help were most appealing, respectively.

Despite the health and economic burden from cannabis use, there is very little research on the sources of support sought by cannabis consumers, including in North America, in which an increasing number of jurisdictions have legalized cannabis. While it is plausible that legalization might increase help-seeking due to reductions in stigma and potential increases in services and support, we are unaware of any studies that have examined differences in help-seeking between jurisdictions with differing cannabis laws. Understanding what services cannabis consumers are most likely to access may have important implications for public health campaigns as they may target specific intervention types to streamline the gap between problematic cannabis use and treatment.

The current study sought to examine the proportion and type of help-seeking behaviours among a population-based sample of cannabis consumers. The study also examined differences in help-seeking by predisposing, enabling/impeding, and need factors.

## 2 Material and methods

### 2.1 Respondents and procedures

Data are cross-sectional findings from the 2022 wave (collected in September-October 2022) of the International Cannabis Policy Study (ICPS), conducted in Canada and the United States. The ICPS surveys, offered in both either English (Canada and US) or French (Canada only) (www.cannabisproject.ca/methods) include a detailed set of questions relating to an individual’s cannabis use frequency, quantity of consumption, types of products consumed, and amount of time since last use. As documented elsewhere (Hammond et al., 2020, Hammond et al., 2022), respondents aged 16–65 were recruited through the Neilson Consumer Insights Global Panel and partner’s panel via a unique email link sent to a random sample of panelists. Respondents completed a self-report online survey (median survey time across past 12-month cannabis consumers was 35.9 minutes). The cooperation rate across all countries was 60.7 %. To ensure data quality and integrity and incorporate best practices for online surveys, attention checks, ‘trap’ questions, and monetary incentives were included to decrease response bias. A full report on the methodology of the ICPS surveys can be found in the Technical Reports and methodology paper (Corsetti et al., 2023, Hammond et al., 2020). The ICPS survey also received ethics approval from the University of Waterloo.

### 2.2 Measures

#### 2.2.1 Socio-demographics

All respondents provided demographic information including age, sex-at-birth, gender identity, sexuality, ethnicity/race, highest education level and perceived income adequacy (i.e., whether they feel as though their household can make ends meet).

#### 2.2.2 Cannabis consumption and problematic use

Problematic use was assessed using the WHO’s Alcohol, Smoking, and Substance Involvement Screening Test (ASSIST) (WHO, 2002). As documented in a previous analysis utilizing ICPS data, the ASSIST measures the risk of developing health, social, legal, or financial problems associated with past 3-month cannabis use along with quantifying an individual’s desire to use cannabis, their inability to meet expectations and control their use, and potential concerns raised by family and friends (Fataar et al., 2023). Scores on the ASSIST range from 0 to 39 and are categorized into 3 different severity categories: low (0−7), moderate (8−26), and high (26 +). The current study set the cutoff for low risk at 7 (as opposed to the cut-off of 4 used in some studies) to capture not only frequency of use, since all respondents were past 12-month cannabis consumers, but also at least one harm associated with use (Fataar et al., 2023, Thake and Davis, 2011, Asbridge et al., 2014).

#### 2.2.3 Legal status of recreational cannabis use

An indicator variable was created to represent the legal status of cannabis at the time of data collection in September 2022: 1) ‘recreational’ cannabis (Canada and 20 states); ‘medical cannabis’ (18 states); and ‘illegal’ (13 states; see Table S1).

#### 2.2.4 Perceived addiction

Respondent’s perceived addiction was measured by answering the question “Do you consider yourself to be addicted to marijuana?” Respondent’s responses could have been *Not addicted at all, A little addicted, Very addicted, Don’t know* or *Refuse to Answer* (refusals were omitted from analyses). This item is associated with other more comprehensive measures of dependence for other addictive substances, such as tobacco, but has not been examined in relation to cannabis (Gomes et al., 2024, Pienkowski et al., 2022, Vogel et al., 2020).

#### 2.2.5 Help-seeking sources

Respondents were asked: “In the past 3 months, have you ever tried to get help to manage problems with your marijuana use?” Respondents who responded yes, were asked about sources of help: “Where did you seek help to manage problems with your marijuana use?” Respondents could select as many options as they like from the following 7 sources: doctor or physician, other healthcare provider (e.g., psychologist, psychiatrist, counselor, etc.), online/website support or information, addiction service or clinic, telephone helpline/support, smartphone application, or family or friends.

### 2.3 Data Analysis

The current analysis included respondents who reported using cannabis in the past 12-months. The total cross-sectional sample of individuals in the 2022 wave in both Canada and the US was 56,344. Of these, 14,059 reported past 12-month cannabis use. After excluding 850 participants with invalid/missing data on sex-at-birth, education status and whether they sought help or not, 13,209 individuals were retained for analyses.

Post-stratification sample weights were constructed based on Canadian and US Census and other population level estimates where respondents are classified into age-by-sex-by-province/state, education and age-by-smoking status. A raking algorithm, calibrated to these groupings, was applied to the full sample of respondents to compute the weights which are rescaled to the sample size for Canada and the US separately.

To achieve the first two aims, descriptive statistics are presented to characterize the number of respondents who endorsed any help-seeking and what sources of help were most likely to be accessed. To test the third aim, a binary logistic regression was run to determine correlates of help-seeking from any of the 7 sources (0 =no help sought, 1 =help sought from at least one source). Finally, to test the fourth aim, a total of 7 binary regression models were run to examine correlates associated with seeking help from each of the 7 sources. A Pearson correlation was run with both the perceived addiction and ASSIST severity scores to determine whether multicollinearity existed. Multicollinearity did not exist (*r* = 0.2) as our correlation did not reach the threshold of 0.8 and consequently both variables are presented in the final models (Obite et al., 2020). Analyses were conducted using SAS Studio.

## 3 Results

### 3.1 Sample characteristics and prevalence of help-seeking among past 12-month consumers

**Fig. 1:**
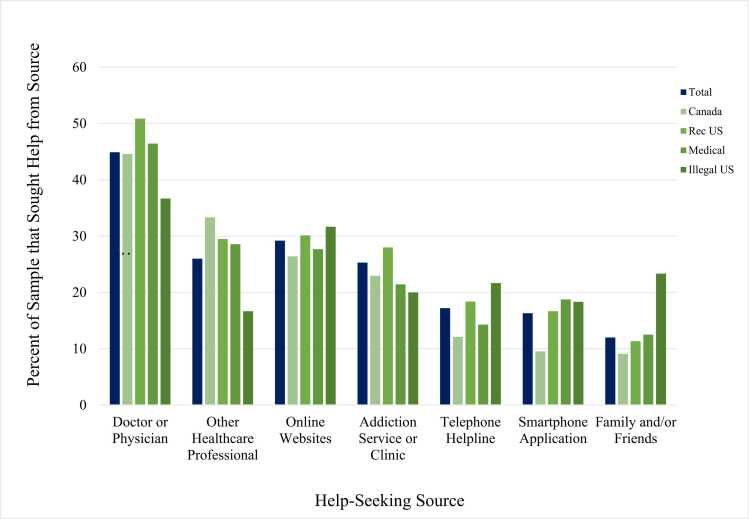
Help-Seeking Sources from Past 12-Month Cannabis Consumers who Sought Help based on Legal Status (*N* = 13,209).

Table 1 shows the sample profile of the 13,209 respondents in the US and Canada. Of the 13,209 respondents who reported using cannabis in the past 12 months, 9.2 % reported seeking some form of help to manage their cannabis use. As Fig. 1 indicates, respondents who sought help were most likely to do so from a doctor or physician (44.9 %), followed by online/website support or information (29.2 %), other healthcare providers (i.e., psychologist, psychiatrist, counselor, etc.; 26.0 %), addiction service or clinic (25.3 %), telephone helplines/support (17.2 %), smartphone app (16.3 %), and lastly, family or friends (12.0 %). Table S2 displays how many people sought each source of help including from multiple sources.

**Table 1:** Demographic characteristics of past 12-month cannabis consumers in wave 5 of the International Cannabis Policy Study (*N* = 13,209).

Characteristics		Unweighted, % (*n*) N = 13,209	Weighted, % (*n*) N = 13,209
Age			
	16–25 years	14.4 % (1900)	17.5 % (2305)
	26–35 years	24.2 % (3194)	28.1 % (3717)
	36–45 years	25.8 % (3413)	23.1 % (3055)
	46–55 years	17.8 % (2344)	17.4 % (2303)
	56–65 years	17.9 % (2358)	13.8 % (1829)
Sex-at-Birth			
	Male	35.1 % (8569)	54.3 % (7175)
	Female	64.9 % (4640)	45.7 % (6034)
Gender Identity			
	Woman	64.6 % (8534)	45.8 % (6043)
	Man	35.1 % (4641)	54.0 % (7136)
	Transgender/Nonbinary/Other	0.13 % (17)	0.11 % (15)
	Don’t know/Refuse to answer	0.13 % (17)	0.11 % (15)
Ethnicity			
	White/Caucasian	77.9 % (10,283)	73.5 % (9711)
	Other/Mixed/Unstated	22.2 % (2926)	26.5 % (3499)
Sexuality			
	Heterosexual/Straight	84.0 % (11,089)	85.9 % (11,341)
	Other	16.1 % (2120)	14.1 % (1868)
Education			
	Less than high school	8.2 % (1086)	12.0 % (1589)
	High School Diploma or Equivalent	21.2 % (2802)	26.4 % (3489)
	Some College/University & Technical Certificate/Apprenticeship	40.4 % (5339)	37.0 % (4886)
	Bachelors Degree or higher	30.2 % (3982)	24.6 % (3246)
Income Adequacy			
	Very Difficult	13.4 % (1769)	12.0 % (1590)
	Difficult	24.3 % (3219)	23.8 % (3139)
	Neither easy nor difficult	33.3 % (4397)	32.4 % (4275)
	Easy	17.5 % (2315)	18.1 % (2385)
	Very Easy	9.5 % (1250)	11.6 % (1531)
	Not Stated	2.0 % (259)	2.2 % (290)
Perceived addiction			
	Not at all Addicted	64.2 % (8476)	59.6 % (7906)
	A Little Addicted	24.0 % (3166)	26.4 % (3492)
	Very Addicted	9.2 % (1213)	11.2 % (1483)
	Don’t Know	2.7 % (354)	2.5 % (328)
ASSIST Categories			
	Low	54.9 % (7257)	49.7 % (6565)
	Moderate	35.8 % (4727)	40.0 % (5279)
	High	9.3 % (1225)	10.3 % (1366)
Legal Status			
	Legal in Canada	26.6 % (3513)	28.3 % (3742)
	Legal in US	52.2 % (6889)	35.2 % (4651)
	Medically legal in US	14.8 % (1949)	20.1 % (2660)
	Illegal in US	6.5 % (858)	16.3 % (2157)

### 3.2 Correlates of any Help-seeking

Table 2 reports that 7 factors were associated with any form of help-seeking. Respondent’s perceived addiction to cannabis was associated with a greater likelihood of seeking help from any help-seeking source: higher perceived addiction was related to more help-seeking versus those who reported not being addicted to cannabis (AOR = 4.94, 95 % CI=3.51,6.96). Problematic cannabis use (ASSIST scores) also predicted help-seeking, where higher severity predicted higher help-seeking than those in the low category (AOR = 4.05, 95 % CI=2.50,6.56). Respondents were also more likely to seek help if they were younger (all contrasts *p* < .03 except between those who were 16–25 and 25–35 years old), reported male sex-at-birth (*p* = .002), and reported their race/ethnicity as something other than white/Caucasian (*p* = .012). Lastly, individuals most likely to seek help from any source reported higher education (all contrasts *p* < .001) and it being very easy to makes ends meet in comparison to those who found it very difficult (*p* = .001). Factors not associated with any type of help-seeking include the legal status of cannabis in the respondent’s jurisdiction (*p* = .554) and their sexuality (*p* = .119).

**Table 2:** Predictors of help-seeking to manage cannabis-related problems among past 12-month cannabis consumers (*N* = 13,209).

Predictor		*Sought help (%)*	*β*	*p*	*AOR*	*95 % OR CI*
Legal Status						
	Canada (Recreational)	8.4 %	0			
	US Recreational	9.9 %	−.0241	.849	0.98	0.76–1.25
	US Medical	9.1 %	−.0153	.926	0.96	0.71–1.36
	US Illegal	9.2 %	−.2849	.157	0.75	0.51–1.12
**Perceived Addiction**						
	Not at all addicted	3.0 %	0			
	A little addicted	13.4 %	.8764	< .001	2.40	1.73–3.35
	Very addicted	32.7 %	1.597	< .001	4.94	3.51–6.96
	Don’t know	6.2 %	.4780	.241	1.61	0.73–3.59
**ASSIST**						
	Low	1.5 %	0			
	Moderate	19.0 %	2.038	< .001	7.68	5.32–11.0
	High	8.1 %	1.399	< .001	4.05	2.50–6.56
**Age**						
	16–25 years	11.1 %	0			
	26–35 years	12.8 %	−.2397	.139	0.79	0.57–1.08
	36–45 years	11.6 %	−.3499	.025	0.71	0.52–0.96
	46–55 years	4.8 %	−.8596	< .001	0.42	0.28–0.64
	56–65 years	0.9 %	−2.161	< .001	0.12	0.06–0.21
**Sex-at-Birth**						
	Male	12.0 %	0			
	Female	5.9 %	−.3586	.002	0.70	0.56–0.88
Sexuality						
	Other	7.3 %	0			
	Heterosexual	9.5 %	.2380	.119	1.27	0.94–1.71
**Ethnicity**						
	Other/Mixed/Unstated	11.2 %	0			
	White/Caucasian	8.4 %	−.3185	.011	0.73	0.57–0.93
**Education**						
	Bachelors degree or higher	6.5 %	0			
	Less than high school	7.3 %	−1.215	< .001	0.30	0.20–0.45
	High School Diploma/ GED	5.8 %	−.7671	< .001	0.46	0.34–0.64
	Some College/University	17.6 %	−.7726	< .001	0.46	0.34–0.62
**Income Adequacy**						
	Very Difficult	9.0 %	0			
	Difficult	4.9 %	−.7034	< .001	0.50	0.33–0.74
	Neither easy nor difficult	5.0 %	−.7554	< .001	0.47	0.32–0.68
	Easy	11.2 %	.0124	.951	1.01	0.68–1.50
	Very Easy	26.8 %	.7419	< .001	2.10	1.40–3.15

*Note: R*^2^ = .36, *F* (23, 13,186) = 104.75, *p* < .001. AOR =  adjusted odds ratio.

### 3.3 Factors Associated with Specific Sources of Help

Binary logistic regressions were run for each of the 7 sources of help to identify factors that predict source-specific help-seeking.

#### 3.3.1 Legal Status of Cannabis

The legal status of cannabis was a significant predictor of help-seeking among 4 different sources of help. Respondents who reside in the legal Canadian market (AOR = 2.17, 95 % CI=1.11, 4.17) or legal US market (AOR = 1.93, 95 % CI=1.01, 3.67) were more likely to seek help from a doctor and/or physician than those who reside in an illegal US market (no differences found between the legal Canadian market, legal US market or medically legal US market; see Table S3). In contrast, Canadian respondents (AOR = 0.57, 95 % CI=0.34, 0.97) and respondents in an illegal US market (AOR = 0.43, 95 % CI=0.19, 0.98) were less likely to seek help from addiction services than those who reside in a legal US market (see Table S4). Canadian respondents (AOR = 0.44, 95 % CI=0.22, 0.85) were also less likely to seek help from a smartphone application than respondents in a legal US market – see Table S5. Lastly, respondents who resided in an illegal US market were much more likely to seek help from family and friends than those who reside in a legal Canadian (AOR = 5.73, 95 % CI=2.21, 14.91), legal US (AOR = 4.76, 95 % CI=2.00, 11.11) and legal medical market in the US (AOR = 3.57, 95 % CI=1.33, 8.33; see Table S6).

#### 3.3.2 Perceived Addiction

Respondent’s who identified themselves as very addicted to cannabis were more likely to seek help from doctors and/or physicians (AOR = 1.91, 95 % CI=1.09, 3.35, see Table S3) and other health care providers such as psychologists and psychiatrists (AOR = 2.30, 95 % CI=1.12, 4.72; see Table S7) in comparison to those who identified themselves as not at all addicted to cannabis.

#### 3.3.3 Cannabis consumption and problematic use

Individuals with low ASSIST scores were much less likely to seek help from online sources (AOR = 0.41, 95 % CI=0.20, 0.87, see Table S8) and an addiction service or clinic (AOR = 0.32, 95 % CI=0.11, 0.95, see Table S4) than those with moderate ASSIST scores.

#### 3.3.4 Age

Respondent’s aged 25–35 were much less likely than any other age group to seek help from a doctor and/or physician (*p* < .02 for all contrasts) and respondent’s who were 36–45 were much less likely to seek help from a psychologist and/or psychiatrist (*p* < .01 for all contrasts) than any other age group except for those aged 56–65.

#### 3.3.5 Sex-at-birth

Sex-at-birth was only associated with help-seeking from an online source where females-at-birth were much less likely to seek help online than males-at-birth (AOR = 0.54, 95 % CI=0.34, 0.87, see Table S8).

#### 3.3.6 Education

Respondents who sought help from either a doctor or physician (AOR = 1.75, 95 % CI=1.03, 3.03; see Table S3) or other healthcare provider (AOR = 2.14, 95 % CI=1.16, 3.85; see Table S7) such as a psychologist or psychiatrist were much more likely to have at minimum, a bachelor’s degree in comparison to those with a high school degree. Additionally, respondents who had completed some college/university, or held a certificate or diploma (AOR = 3.45, 95 % CI=1.45, 8.33) or had at minimum, a bachelor’s degree (AOR = 2.50, 95 % CI=1.04, 5.88), were much more likely to seek help from an addiction service or clinic than those without a high school diploma (see Table S4). Similarly, those without a high school diploma were also less likely to seek help through a telephone helpline than those with a bachelor’s degree (AOR = 0.36, 95 % CI=0.13, 0.99) and were also less likely to seek help through a smartphone application than those with a high school diploma (AOR = 0.19, 95 % CI=0.05, 0.78) or some college/technical training (AOR = 0.17, 95 % CI=0.04, 0.70).

#### 3.3.7 Income adequacy

Those who indicated that it is very easy to make ends meet were much more likely to seek help from a doctor or physician (AOR = 1.92, 95 % CI=1.06, 3.45) and family and friends (AOR = 0.33, 95 % CI=0.12, 0.89) than respondent’s who reported it was neither easy or difficult to make ends meet. Similarly, those who found it easy to make ends meet were more likely than respondents who found it neither easy nor difficult to make ends meet to seek help from a smartphone application (AOR = 3.19, 95 % CI=1.28, 7.69). That said, respondent’s who found it very difficult to make ends meet were much more likely to seek help from an addiction service or clinic than those who found it very easy (AOR = 2.27, 95 % CI=1.09, 4.76) to make ends meet (see Table S6).

## 4 Discussion

The current study is among the most comprehensive efforts to examine treatment-seeking and other sources of help among national samples of cannabis consumers. Overall, approximately 1 in 10 cannabis consumers in the US and Canada reported seeking help for their cannabis use in the past three months. This finding is similar to past research reporting that 10 % of daily or near-daily cannabis consumers seek treatment (Sherman and McRae-Clark, 2016). The findings also align with national estimates from the 2022 Canadian Cannabis Survey, which report that 3 % of individuals who have ever used cannabis in their lifetime report seeking professional help for cannabis-related problems (Government of Canada, 2022). Doctors or physicians were the most common source of past 3-month help-seeking highlighting the need for them to be well educated on how to deal with cannabis-related questions and have resources available to support patients wishing to reduce their cannabis related consequences. Since doctors serve as the main source of contact for cannabis-related problems, its speculated that individuals may feel most comfortable discussing their cannabis use with their primary care provider and asking for advice prior to initiating other more formal sources of help (e.g., psychologists or psychiatrists or addiction services). In addition, doctors/physicians are covered under Canadian healthcare and basic health insurance programs for individuals in the US, thereby making them the most accessible provider to discuss concerns with.

Online websites were the second most common source of help, highlighting that sources of information that are confidential, accessible, and free to the public are being accessed frequently and should provide accurate information regarding cannabis related harms and resources for consumers who want further assistance. Online sources may serve as a critical first step for those determining whether they have a real issue with their cannabis use and there is a need for easy to digest, readily accessible resources on problematic cannabis use and cannabis use disorder treatment. While most research in this field focuses on the barriers that hinder consumers from seeking help from formal addiction clinics and treatment programs, the current study highlights that research should broaden this scope as cannabis consumers are frequently accessing doctors and physicians and other, more accessible sources of information, such as resources online.

Below, the specific factors of help-seeking are broken down into their three categories and similarities and differences from past literature are discussed.

### 4.1 Enabling/imposing factors

The enabling/imposing factors that were assessed in the current study was the legal status of cannabis in the respondent’s jurisdiction, education level, and income adequacy. In jurisdictions with legal ‘recreational’ markets, respondents were more likely to seek help from their doctor or physician in comparison to respondents in jurisdictions where cannabis is illegal suggesting that legalization may have made individuals feel more comfortable talking about their cannabis-related problems to their doctor. In contrast, respondents living in states where recreational cannabis had not been legalized were more likely to seek help from family and friends, which may reflect greater reliance on informal sources of support due to lower comfort levels discussing cannabis with physicians. However, differences by legal status/jurisdictions were modest, which may reflect relatively similar levels of social norms towards cannabis regardless of jurisdiction.

The current study is consistent with findings from a national survey in Ireland, in which those who were seeking formal addiction treatment for cannabis use were more likely to be unemployed and have no (or limited) educational attainment (Mongan et al., 2022). That said, the current findings extend beyond help-seeking from formal addiction services and suggest that those who were more financially stable were much more likely to seek help from a doctor or a smartphone application than those who were less financially stable. Future research will benefit from understanding why individuals with lower socioeconomic statuses are less likely to seek help from doctors or physicians and more likely to seek help from addiction treatment programs.

### 4.2 Need factors

The two main need factors that were assessed in the current study were perceived addiction and ASSIST severity scores. The presence of the perceived addiction variable did not impact the likelihood of ASSIST scores predicting variance in the models which was interesting as it has been identified as a barrier to treatment (Zvolensky et al., 2018) and a predictor of treatment seeking (Kerridge et al., 2017, Van Der Pol et al., 2013). Perceived addiction and ASSIST scores continue to be significant predictors of any type of help seeking (Papinczak et al., 2017). This may indicate that wider adoption of screening by physicians, such as using the ASSIST tool or asking patients whether they believe they have a problem with cannabis, may improve help-seeking from problematic cannabis consumers. Specifically for doctors/physicians and other medical professionals it was found that those who perceived themselves as more addicted to cannabis were more likely to seek help from these sources than those who indicated that they did not know or were not addicted to cannabis. Consequently, respondent’s who recognize they have problems with their cannabis use are more likely to seek help.

### 4.3 Predisposing factors

The predisposing factors that were assessed in the current study were age, sex-at-birth, sexuality, ethnicity/race, and education level. Among these, age was the most common predictor of help-seeking, suggesting that respondents who were younger were more likely to seek help from doctors and other healthcare professionals than older respondents. Past research has found that those in the age group of 18–25 were much less likely to utilize formal cannabis use disorder treatment (i.e., addiction treatment services) than those who were over the age of 26 (Askari, Keyes and Mauro, 2021). In contrast, though age was not a significant predictor of addiction treatment services in the current study, it provides a more nuanced understanding of the different age groups seeking help via different sources.

### 4.4 Limitations

Although the ICPS data is among the largest population-based studies of cannabis use, few consumers reported seeking help for cannabis related problems from each of the sources of help; consequently, each of the regression models had limited statistical power to examine correlates of treatment seeking for each individual source of help. Future research would also benefit from distinguishing between what types of problems individuals were having when seeking help from each of the sources, their level of engagement with each source, and clarifying that it was the respondent’s choice to initiate seeking help (not a doctor initiating discourse about the patient’s cannabis-related problems, leading them to seek assistance). Further, future surveys should ascertain where help-seekers who sought help from more than one source, sought help from first. In addition, future research should determine why respondent’s aged 18–25 were more likely to seek help and why older adults would be less likely to seek help. The cross-sectional nature of this study also limits our ability to determine causality. For instance, reporting that a relationship exists between respondents perceived addiction and help-seeking does not imply that respondents are seeking help due to their ability to recognize they have a problem; instead, they may learn they have a problem from seeking help.

## 5 Conclusion

Help-seekers were most likely to seek help from their doctor or physician and online resources. Few differences in help-seeking were observed among consumers living in states that had not legalized ‘recreational’ cannabis. The findings highlight the need to examine population-based approaches for supporting cannabis consumers experiencing problematic patterns of use, particularly cost-effective, easily accessible sources that minimize barriers to help-seeking.

## Funding acknowledgment

Funding for the ICPS study was provided by a Canadian Institutes of Health Research Project Bridge Grant (PJT-153342) and a Canadian Institutes of Health Research Project Grant (PJT-153342).

## Declaration interests

DH has provided paid expert testimony on behalf of public health authorities in response to legal claims from the tobacco, vaping and cannabis industry. All remaining authors declare no conflicts of interest. The funders played no role in the conceptualization, analysis or interpretation of the study findings.

## CRediT authorship contribution statement

**David Hammond:** Writing – review & editing, Validation, Supervision, Software, Resources, Project administration, Methodology, Investigation, Funding acquisition, Conceptualization. **Samantha Rundle:** Writing – review & editing, Writing – original draft, Visualization, Validation, Project administration, Investigation, Formal analysis, Data curation, Conceptualization.

## Declaration of Competing Interest

none.
