# Supplementary material for: Help-seeking behaviours among cannabis consumers in Canada and the United States: Findings from the international cannabis policy study
Source: Drug Alcohol Depend Rep. 2024 Dec 11;14:100306. doi: 10.1016/j.dadr.2024.100306 (PMC11732192; doi:10.1016/j.dadr.2024.100306)
Supplement: Supplementary file 1 — Supplementary material [file mmc1.docx]

Supplemental Table 1. Number of U.S. States in each of the legal status categories.

| Legal Status of Cannabis | Number of States |
| --- | --- |
| **Recreationally Legal** | 20 |
| **Medically Legal** | 18 |
| **Illegal** | 13 |

Supplemental Table 2. Number of respondents who sought help from each source (N = 1,143).

|  | Number of Sources Sought | | | | | | |
| --- | --- | --- | --- | --- | --- | --- | --- |
| Help-Seeking Options | One | Two | Three | Four | Five | Six | Seven |
| Doctor | 239 | 139 | 91 | 47 | 15 | 13 | 1 |
| Other Healthcare | 103 | 100 | 79 | 23 | 4 | 6 | 1 |
| Online | 67 | 108 | 115 | 41 | 10 | 11 | 1 |
| Addiction | 58 | 108 | 72 | 39 | 16 | 12 | 1 |
| Telephone | 26 | 63 | 62 | 30 | 13 | 13 | 1 |
| Smartphone | 47 | 63 | 42 | 19 | 15 | 10 | 1 |
| Family/Friends | 53 | 25 | 22 | 24 | 8 | 11 | 1 |

Note: Respondents who sought help from two or more sources are accounted for n number of times per source sought (e.g., there were 303 respondents who sought help from two sources, therefore they are represented twice in the ‘two’ column).

Supplemental Table 3. Predictors of help-seeking from a doctor or physician to manage cannabis-related problems among past 12-month cannabis consumers (*N* = 13,209).

| Predictor |  | *Sought Help (%)* | *β* | *p* | *AOR* | *95% AOR CI* |
| --- | --- | --- | --- | --- | --- | --- |
| **Legal Status** | |  |  |  |  |  |
|  | Canada (Recreational) | 3.8% | 0 |  |  |  |
|  | US Recreational | 4.7% | -.1200 | .605 | 0.89 | 0.56-1.40 |
|  | US Medical | 4.1% | -.2677 | .403 | 0.77 | 0.41-1.43 |
|  | US Illegal | 3.4% | -.7754 | .024 | 0.46 | 0.24-0.90 |
| **Perceived Addiction** | |  |  |  |  |  |
|  | Not at all addicted | 1.1% | 0 |  |  |  |
|  | A little addicted | 5.3% | .1234 | .659 | 1.13 | 0.65-1.96 |
|  | Very addicted | 17.8% | .6446 | .025 | 1.91 | 1.09-3.45 |
|  | Don’t know | 3.4% | 1.134 | .112 | 3.11 | 0.77-12.6 |
| ASSIST |  |  |  |  |  |  |
|  | Low | 0.6% | 0 |  |  |  |
|  | Moderate | 8.9% | .0059 | .985 | 1.01 | 0.54-1.88 |
|  | High | 2.7% | -.4165 | .379 | 0.66 | 0.26-1.67 |
| **Age** |  |  |  |  |  |  |
|  | 16-25 years | 5.0% | 0 |  |  |  |
|  | 26-35 years | 4.2% | -.7517 | .005 | 0.47 | 0.28-0.80 |
|  | 36-45 years | 6.9% | .1675 | .527 | 1.18 | 0.70-1.99 |
|  | 46-55 years | 2.5% | .1215 | .756 | 1.13 | 0.53-2.43 |
|  | 56-65 years | 0.3% | -.7700 | .282 | 0.46 | 0.11-1.89 |
| Sex-at-Birth |  |  |  |  |  |  |
|  | Male | 5.8% | 0 |  |  |  |
|  | Female | 2.2% | -.1840 | .389 | 0.83 | 0.55-1.27 |
| Sexuality |  |  |  |  |  |  |
|  | Other | 3.4% | 0 |  |  |  |
|  | Heterosexual | 4.2% | -.1524 | .594 | 0.86 | 0.49-1.50 |
| Ethnicity |  |  |  |  |  |  |
|  | Other/Mixed/Unstated | 4.2% | 0 |  |  |  |
|  | White/Caucasian | 4.1% | .2796 | .208 | 1.32 | 0.86-2.05 |
| **Education** |  |  |  |  |  |  |
|  | Bachelors degree or higher | 2.8% | 0 |  |  |  |
|  | Less than high school | 2.5% | -.2421 | .541 | 0.79 | 0.36-1.71 |
|  | High School Diploma/ GED | 2.0% | -.5706 | .040 | 0.57 | 0.33-0.97 |
|  | Some College/University | 9.8% | -.5300 | .053 | 0.59 | 0.34-1.01 |
| Income Adequacy | |  |  |  |  |  |
|  | Very Difficult | 3.9% | 0 |  |  |  |
|  | Difficult | 1.7% | -.1916 | .637 | 0.83 | 0.37-1.83 |
|  | Neither easy nor difficult | 1.8% | -.3056 | .396 | 0.74 | 0.36-1.49 |
|  | Easy | 4.7% | -.1744 | .623 | 0.84 | 0.42-1.69 |
|  | Very Easy | 15.5% | .3490 | .326 | 1.42 | 0.71-2.85 |

*Note:* *R*^2^ = .18, *F* (23, 848) = 5.45, *p* < .001. AOR = adjusted odds ratio.

Supplemental Table 4. Predictors of help-seeking from an addiction service or clinic to manage cannabis-related problems among past 12-month cannabis consumers (*N* = 13,209).

| Predictor |  | *Sought Help (%)* | *β* | *p* | *AOR* | *95% AOR CI* |
| --- | --- | --- | --- | --- | --- | --- |
| **Legal Status** | |  |  |  |  |  |
|  | Canada (Recreational) | 1.7% | 0 |  |  |  |
|  | US Recreational | 3.2% | .5572 | .039 | 1.75 | 1.03-2.97 |
|  | US Medical | 2.1% | .1405 | .705 | 1.15 | 0.56-2.38 |
|  | US Illegal | 1.8% | -.2872 | .508 | 0.75 | 0.32-1.76 |
| **Perceived Addiction** | |  |  |  |  |  |
|  | Not at all addicted | 0.5% | 0 |  |  |  |
|  | A little addicted | 3.6% | .6089 | .107 | 1.84 | 0.88-3.86 |
|  | Very addicted | 8.9% | .5880 | .113 | 1.80 | **0.87-3.73** |
|  | Don’t know | 2.4% | 1.725 | .016 | 5.61 | 1.38-22.8 |
| **ASSIST** |  |  |  |  |  |  |
|  | Low | 0.2% | 0 |  |  |  |
|  | Moderate | 5.3% | 1.126 | .041 | 3.08 | 1.05-9.05 |
|  | High | 1.3% | .3715 | .597 | 1.45 | 0.37-5.75 |
| Age |  |  |  |  |  |  |
|  | 16-25 years | 2.4% | 0 |  |  |  |
|  | 26-35 years | 3.8% | -.0064 | .983 | 0.99 | 0.55-1.80 |
|  | 36-45 years | 2.7% | -.2535 | .427 | 0.78 | 0.42-1.45 |
|  | 46-55 years | 1.0% | -.3973 | .400 | 0.67 | 0.27-1.70 |
|  | 56-65 years | 0.2% | -.2525 | .729 | 0.78 | 0.19-3.25 |
| Sex-at-Birth | |  |  |  |  |  |
|  | Male | 3.2% | 0 |  |  |  |
|  | Female | 1.2% | -.3579 | .170 | 0.70 | 0.42-1.17 |
| Sexuality |  |  |  |  |  |  |
|  | Other | 1.8% | 0 |  |  |  |
|  | Heterosexual | 2.4% | .0065 | .984 | 1.01 | 0.53-1.92 |
| Ethnicity |  |  |  |  |  |  |
|  | Other/Mixed/Unstated | 2.2% | 0 |  |  |  |
|  | White/Caucasian | 2.4% | .4895 | .073 | 1.63 | 0.96-2.79 |
| **Education** |  |  |  |  |  |  |
|  | Bachelors degree or higher | 0.8% | 0 |  |  |  |
|  | Less than high school | 1.4% | -.9089 | .041 | 0.40 | 0.17-0.96 |
|  | High School Diploma or GED | 1.9% | -.3540 | .343 | 0.70 | 0.34-1.46 |
|  | Some College/University | 4.9% | .3196 | .317 | 1.38 | 0.74-2.58 |
| **Income Adequacy** | |  |  |  |  |  |
|  | Very Difficult | 3.0% | 0 |  |  |  |
|  | Difficult | 1.4% | -.3764 | .378 | 0.69 | 0.30-1.59 |
|  | Neither easy nor difficult | 1.2% | -.4113 | .320 | 0.66 | 0.29-1.49 |
|  | Easy | 2.8% | -.5261 | .199 | 0.59 | 0.27-1.32 |
|  | Very Easy | 5.9% | -.8162 | .030 | 0.44 | 0.21-0.92 |

*Note*: *R*^2^ = .12, *F* (23, 848) = 3.18, *p* < .001. AOR = adjusted odds ratio.

Supplemental Table 5. Predictors of help-seeking from a smartphone application to manage cannabis-related problems among past 12-month cannabis consumers (*N* = 13,209).

| Predictor | |  | *Sought Help (%)* | *β* | *p* | *AOR* | *95% AOR CI* |
| --- | --- | --- | --- | --- | --- | --- | --- |
| **Legal Status** | | |  |  |  |  |  |
|  | Canada (Recreational) | | 0.8% | 0 |  |  |  |
|  | US Recreational | | 1.9% | .8342 | .014 | 2.30 | 1.18-4.48 |
|  | US Medical | | 1.6% | .6703 | .109 | 1.96 | 0.86-4.44 |
|  | US Illegal | | 1.9% | .9175 | .058 | 2.50 | 0.97-6.45 |
| Perceived Addiction | | |  |  |  |  |  |
|  | Not at all addicted | | 0.5% | 0 |  |  |  |
|  | A little addicted | | 1.9% | -.0910 | .826 | 0.91 | 0.40-2.06 |
|  | Very addicted | | 6.2% | .3121 | .440 | 1.37 | 0.92-3.02 |
|  | Don’t know | | 0.1% | -2.247 | .060 | 0.11 | 0.01-1.10 |
| ASSIST |  | |  |  |  |  |  |
|  | Low | | 0.2% | 0 |  |  |  |
|  | Moderate | | 3.1% | -.2976 | 540 | 0.74 | 0.29-1.93 |
|  | High | | 1.3% | -.1747 | .800 | 0.84 | 0.22-3.22 |
| Age |  | |  |  |  |  |  |
|  | 16-25 years | | 1.6% | 0 |  |  |  |
|  | 26-35 years | | 2.7% | .2893 | .419 | 1.34 | 0.66-2.70 |
|  | 36-45 years | | 1.6% | -.1968 | .620 | 0.82 | 0.38-1.79 |
|  | 46-55 years | | 0.5% | -.7564 | .242 | 0.47 | 0.13-1.67 |
|  | 56-65 years | | 0.1% | -.7901 | .383 | 0.45 | 0.08-2.68 |
| Sex-at-Birth |  | |  |  |  |  |  |
|  | Male | | 2.1% | 0 |  |  |  |
|  | Female | | 0.8% | -.1934 | .513 | 0.82 | 0.46-1.47 |
| Sexuality |  | |  |  |  |  |  |
|  | Other | | 0.9% | 0 |  |  |  |
|  | Heterosexual | | 1.6% | .2295 | .554 | 1.26 | 0.59-2.69 |
| Ethnicity |  | |  |  |  |  |  |
|  | Other/Mixed/Unstated | | 2.1% | 0 |  |  |  |
|  | White/Caucasian | | 1.3% | -.4288 | .164 | 0.65 | 0.36-1.19 |
| Education |  | |  |  |  |  |  |
|  | Bachelors degree or higher | | 0.3% | 0 |  |  |  |
|  | Less than high school | | 1.5% | -1.033 | .176 | 0.36 | 0.08-1.59 |
|  | High School Diploma or GED | | 1.2% | .6218 | .140 | 1.86 | 0.82-4.25 |
|  | Some College/University | | 2.6% | .7345 | .069 | 2.08 | 0.95-4.60 |
| Income Adequacy | | |  |  |  |  |  |
|  | Very Difficult | | 1.2% | 0 |  |  |  |
|  | Difficult | | 0.7% | -.2221 | .720 | 0.80 | 0.24-2.69 |
|  | Neither easy nor difficult | | 0.5% | -.5087 | .319 | 0.60 | 0.22-1.64 |
|  | Easy | | 2.4% | .6507 | .153 | 1.92 | 0.79-4.68 |
|  | Very Easy | | 5.1% | .3833 | .415 | 1.47 | 0.58-3.69 |

*Note*: *R*^2^ = .12, *F* (23, 848) = 2.86, *p* < .001. AOR is an adjusted odds ratio.Supplemental Table 6. Predictors of help-seeking from family and friends to manage cannabis-related problems among past 12-month cannabis consumers (*N* = 13,209).

| Predictor | |  | *Sought Help (%)* | *β* | *p* | *AOR* | *95% AOR CI* |
| --- | --- | --- | --- | --- | --- | --- | --- |
| **Legal Status** | | |  |  |  |  |  |
|  | Canada (Recreational) | | 0.7% | 0 |  |  |  |
|  | US Recreational | | 0.8% | .1820 | .627 | 1.20 | 0.58-2.50 |
|  | US Medical | | 0.9% | .4832 | .333 | 1.62 | 0.61-4.32 |
|  | US Illegal | | 2.5% | 1.746 | < .001 | 5.73 | 2.21-14.9 |
| Perceived Addiction | | |  |  |  |  |  |
|  | Not at all addicted | | 0.5% | 0 |  |  |  |
|  | A little addicted | | 1.4% | -.3857 | .341 | 0.68 | 0.31-1.51 |
|  | Very addicted | | 3.8% | -.0729 | .856 | 0.93 | 0.42-2.05 |
|  | Don’t know | | 0.7% | -.9045 | .399 | 0.41 | 0.05-3.02 |
| ASSIST |  | |  |  |  |  |  |
|  | Low | | 0.2% | 0 |  |  |  |
|  | Moderate | | 2.2% | -.298 | .505 | 0.74 | 0.31-1.79 |
|  | High | | 1.2% | -.202 | .748 | 0.82 | 0.24-2.79 |
| Age |  | |  |  |  |  |  |
|  | 16-25 years | | 1.4% | 0 |  |  |  |
|  | 26-35 years | | 1.3% | -.4144 | .276 | 0.66 | 0.31-1.39 |
|  | 36-45 years | | 1.4% | .0370 | .927 | 1.04 | 0.47-2.29 |
|  | 46-55 years | | 0.7% | .0222 | .967 | 1.02 | 0.35-2.97 |
|  | 56-65 years | | 0.2% | .7980 | .290 | 2.22 | 0.51-9.74 |
| Sex-at-Birth | | |  |  |  |  |  |
|  | Male | | 1.5% | 0 |  |  |  |
|  | Female | | 0.7% | -.4526 | .177 | 0.64 | 0.33-1.23 |
| Sexuality |  | |  |  |  |  |  |
|  | Other | | 0.9% | 0 |  |  |  |
|  | Heterosexual | | 1.1% | -.1756 | .740 | 0.84 | 0.30-2.37 |
| Ethnicity |  | |  |  |  |  |  |
|  | Other/Mixed/Unstated | | 1.4% | 0 |  |  |  |
|  | White/Caucasian | | 1.0% | -.0026 | .994 | 1.00 | 0.51-1.97 |
| Education |  | |  |  |  |  |  |
|  | Bachelors degree or higher | | 0.8% | 0 |  |  |  |
|  | Less than high school | | 0.7% | .4583 | .416 | 1.58 | 0.52-4.78 |
|  | High School Diploma or GED | | 1.1% | .4200 | .372 | 1.52 | 0.61-3.83 |
|  | Some College/University | | 1.6% | .8805 | .066 | 2.41 | 0.94-6.18 |
| Income Adequacy | | |  |  |  |  |  |
|  | Very Difficult | | 1.3% | 0 |  |  |  |
|  | Difficult | | 0.6% | -.0276 | .965 | 0.97 | 0.29-3.28 |
|  | Neither easy nor difficult | | 0.9% | .5184 | .311 | 1.68 | 0.62-4.58 |
|  | Easy | | 1.2% | -.1498 | .766 | 0.86 | 0.32-2.32 |
|  | Very Easy | | 2.5% | -.5864 | .273 | 0.56 | 0.20-1.59 |

*Note*: *R*^2^ = .15, *F* (23, 848) = 3.11, *p* < .001. AOR = adjusted odds ratio.

Supplemental Table 7. Predictors of help-seeking from another health professional to manage cannabis-related problems among past 12-month cannabis consumers (*N* = 13,209).

| Predictor |  | *Sought Help (%)* | *β* | *p* | *AOR* | *95% AOR CI* |
| --- | --- | --- | --- | --- | --- | --- |
| Legal Status | |  |  |  |  |  |
|  | Canada (Recreational) | 2.5% | 0 |  |  |  |
|  | US Recreational | 2.7% | -.0704 | .776 | 0.93 | 0.57-1.51 |
|  | US Medical | 2.3% | -.1985 | .529 | 0.82 | 0.44-1.52 |
|  | US Illegal | 1.7% | -.6409 | .115 | 0.53 | 0.24-1.17 |
| **Perceived Addiction** | |  |  |  |  |  |
|  | Not at all addicted | 0.5% | 0 |  |  |  |
|  | A little addicted | 3.5% | .4595 | .207 | 1.58 | 0.78-3.23 |
|  | Very addicted | 9.9% | .8309 | .024 | 2.30 | 1.12-4.72 |
|  | Don’t know | 1.2% | .3435 | .673 | 1.41 | 0.29-6.95 |
| ASSIST |  |  |  |  |  |  |
|  | Low | 0.5% | 0 |  |  |  |
|  | Moderate | 4.9% | -.6149 | .073 | 0.54 | 0.28-1.06 |
|  | High | 1.9% | -.6283 | .247 | 0.53 | 0.18-1.55 |
| **Age** |  |  |  |  |  |  |
|  | 16-25 years | 3.4% | 0 |  |  |  |
|  | 26-35 years | 3.6% | -.1414 | .601 | 0.87 | 0.51-1.48 |
|  | 36-45 years | 2.1% | -.8520 | .005 | 0.43 | 0.24-0.77 |
|  | 46-55 years | 1.7% | .1567 | .696 | 1.17 | 0.53-2.57 |
|  | 56-65 years | 0.2% | -.3552 | .655 | 0.70 | 0.15-3.33 |
| Sex-at-Birth | |  |  |  |  |  |
|  | Male | 3.0% | 0 |  |  |  |
|  | Female | 1.7% | .3494 | .145 | 1.42 | 0.89-2.27 |
| Sexuality |  |  |  |  |  |  |
|  | Other | 1.6% | 0 |  |  |  |
|  | Heterosexual | 2.5% | .3916 | .221 | 1.48 | 0.79-2.77 |
| Ethnicity |  |  |  |  |  |  |
|  | Other/Mixed/Unstated | 3.1% | 0 |  |  |  |
|  | White/Caucasian | 2.1% | -.1082 | .647 | 0.90 | 0.57-1.43 |
| **Education** |  |  |  |  |  |  |
|  | Bachelors degree or higher | 1.7% | 0 |  |  |  |
|  | Less than high school | 1.5% | -.3896 | .314 | 0.68 | 0.32-1.45 |
|  | High School Diploma/ GED | 1.4% | -.7588 | .014 | 0.47 | 0.26-0.86 |
|  | Some College/University | 5.0% | -.4556 | .207 | 0.63 | 0.31-1.29 |
| Income Adequacy | |  |  |  |  |  |
|  | Very Difficult | 2.3% | 0 |  |  |  |
|  | Difficult | 1.3% | .0709 | .859 | 1.07 | 0.49-2.35 |
|  | Neither easy nor difficult | 1.4% | .0605 | .883 | 1.06 | 0.47-2.38 |
|  | Easy | 3.1% | -.0014 | .997 | 1.00 | 0.49-2.03 |
|  | Very Easy | 6.6% | -.0953 | .786 | 0.91 | 0.46-1.81 |

*Note:* *R*^2^ = .08, *F* (23, 848) = 2.20, *p* = .001. AOR = adjusted odds ratio.

Supplemental Table 8. Predictors of help-seeking from an online website to manage cannabis-related problems among past 12-month cannabis consumers (*N* =13,209).

| Predictor | |  | *Sought Help (%)* | *β* | *p* | *AOR* | *95% AOR CI* |
| --- | --- | --- | --- | --- | --- | --- | --- |
| Legal Status | | |  |  |  |  |  |
|  | Canada (Recreational) | | 2.0% | 0 |  |  |  |
|  | US Recreational | | 3.0% | .0767 | .769 | 1.08 | 0.65-1.80 |
|  | US Medical | | 2.8% | .1900 | .553 | 1.21 | 0.65-2.27 |
|  | US Illegal | | 3.1% | .3208 | .403 | 1.38 | 0.65-2.93 |
| Perceived Addiction | | |  |  |  |  |  |
|  | Not at all addicted | | 0.8% | 0 |  |  |  |
|  | A little addicted | | 3.8% | .0706 | .831 | 1.07 | 0.56-2.06 |
|  | Very addicted | | 10.9% | .1687 | .613 | 1.18 | 0.62-2.28 |
|  | Don’t know | | 0.1% | -2.616 | .021 | 0.07 | 0.01-0.68 |
| **ASSIST** |  | |  |  |  |  |  |
|  | Low | | 0.2% | 0 |  |  |  |
|  | Moderate | | 6.1% | .9003 | .021 | 2.46 | 1.15-5.27 |
|  | High | | 1.4% | .1148 | .844 | 1.12 | 0.36-3.52 |
| Age |  | |  |  |  |  |  |
|  | 16-25 years | | 3.0% | 0 |  |  |  |
|  | 26-35 years | | 3.9% | -.2397 | .385 | 0.79 | 0.46-1.35 |
|  | 36-45 years | | 3.2% | -.4813 | .106 | 0.62 | 0.35-1.11 |
|  | 46-55 years | | 1.6% | -.0507 | .909 | 0.95 | 0.40-2.27 |
|  | 56-65 years | | 0.1% | -1.369 | .095 | 0.25 | 0.05-1.27 |
| **Sex-at-Birth** |  | |  |  |  |  |  |
|  | Male | | 3.9% | 0 |  |  |  |
|  | Female | | 1.2% | -.6164 | .011 | 0.54 | 0.34-0.87 |
| Sexuality |  | |  |  |  |  |  |
|  | Other | | 2.3% | 0 |  |  |  |
|  | Heterosexual | | 2.7% | -.3225 | .276 | 0.72 | 0.41-1.29 |
| Ethnicity |  | |  |  |  |  |  |
|  | Other/Mixed/Unstated | | 2.5% | 0 |  |  |  |
|  | White/Caucasian | | 2.7% | .4737 | .068 | 1.61 | 0.97-2.67 |
| Education |  | |  |  |  |  |  |
|  | Bachelors degree or higher | | 1.2% | 0 |  |  |  |
|  | Less than high school | | 1.7% | -.7373 | .088 | 0.48 | 0.21-1.12 |
|  | High School Diploma or GED | | 1.6% | -.1791 | .589 | 0.84 | 0.44-1.60 |
|  | Some College/University | | 6.1% | -.1321 | .685 | 0.88 | 0.46-1.66 |
| Income Adequacy | | |  |  |  |  |  |
|  | Very Difficult | | 2.7% | 0 |  |  |  |
|  | Difficult | | 1.2% | -.1191 | .800 | 0.90 | 0.35-2.23 |
|  | Neither easy nor difficult | | 1.4% | .1389 | .734 | 1.15 | 0.52-2.56 |
|  | Easy | | 3.0% | -.2755 | .493 | 0.76 | 0.35-1.67 |
|  | Very Easy | | 9.1% | -.0484 | .902 | 0.95 | 0.44-2.06 |

*Note*: *R*^2^ = .12, *F* (23, 848) = 3.14, *p* < .001. AOR = adjusted odds ratio.

Supplemental Table 9. Predictors of help-seeking from a telephone helpline to manage cannabis-related problems among past 12-month cannabis consumers (*N* = 13,209).

| Predictor | |  | *Sought Help (%)* | *β* | *p* | *AOR* | *95% AOR CI* |
| --- | --- | --- | --- | --- | --- | --- | --- |
| Legal Status | | |  |  |  |  |  |
|  | Canada (Recreational) | | 0.9% | 0 |  |  |  |
|  | US Recreational | | 1.8% | .2706 | .386 | 1.31 | 0.71-2.42 |
|  | US Medical | | 1.7% | .4733 | .236 | 1.61 | 0.73-3.51 |
|  | US Illegal | | 2.1% | .5307 | .281 | 1.70 | 0.65-4.47 |
| Perceived Addiction | | |  |  |  |  |  |
|  | Not at all addicted | | 0.5% | 0 |  |  |  |
|  | A little addicted | | 2.3% | .0637 | .878 | 1.07 | 0.47-2.41 |
|  | Very addicted | | 6.2% | .0887 | .829 | 1.09 | 0.49-2.45 |
|  | Don’t know | | 0.5% | -.9134 | .417 | 0.40 | 0.04-3.66 |
| **ASSIST** |  | |  |  |  |  |  |
|  | Low | | 0.1% | 0 |  |  |  |
|  | Moderate | | 3.4% | 2.699 | <.001 | 1.37 | 3.56-62.1 |
|  | High | | 2.0% | 3.195 | <.001 | 14.9 | 4.80-124 |
| Age |  | |  |  |  |  |  |
|  | 16-25 years | | 1.5% | 0 |  |  |  |
|  | 26-35 years | | 2.5% | .0533 | .875 | 1.02 | 0.31-3.38 |
|  | 36-45 years | | 2.0% | -.2111 | .563 | 1.29 | 0.49-3.41 |
|  | 46-55 years | | 0.9% | -.1380 | .788 | 0.67 | 0.25-1.81 |
|  | 56-65 years | | 0.1% | -1.112 | .265 | 0.90 | 0.34-2.34 |
| Sex-at-Birth | | |  |  |  |  |  |
|  | Male | | 2.3% | 0 |  |  |  |
|  | Female | | 0.8% | -.2390 | .391 | 0.79 | 0.46-1.36 |
| Sexuality |  | |  |  |  |  |  |
|  | Other | | 0.8% | 0 |  |  |  |
|  | Heterosexual | | 1.7% | .3112 | .470 | 1.37 | 0.59-3.18 |
| Ethnicity |  | |  |  |  |  |  |
|  | Other/Mixed/Unstated | | 1.5% | 0 |  |  |  |
|  | White/Caucasian | | 1.6% | .2436 | .414 | 1.28 | 0.71-2.29 |
| **Education** |  | |  |  |  |  |  |
|  | Bachelors degree or higher | | 0.5% | 0 |  |  |  |
|  | Less than high school | | 0.8% | -1.036 | .048 | 0.36 | 0.13-0.90 |
|  | High School Diploma or GED | | 1.0% | -.5502 | .197 | 0.58 | 0.25-1.33 |
|  | Some College/University | | 3.8% | -.2476 | .504 | 0.78 | 0.38-1.62 |
| Income Adequacy | | |  |  |  |  |  |
|  | Very Difficult | | 1.6% | 0 |  |  |  |
|  | Difficult | | 0.8% | .0146 | .981 | 1.02 | 0.31-3.38 |
|  | Neither easy nor difficult | | 1.0% | .2552 | .607 | 1.29 | 0.49-3.41 |
|  | Easy | | 1.5% | -.4028 | .428 | 0.67 | 0.25-1.81 |
|  | Very Easy | | 5.4% | -.1084 | .824 | 0.90 | 0.34-2.34 |

*Note*: *R*^2^ = .10, *F* (23, 848) = 2.41, *p* < .001. AOR = adjusted odds ratio.
